# Atherosclerosis, Dyslipidemia, and Inflammation: The Significant Role of Polyunsaturated Fatty Acids

**DOI:** 10.1155/2013/191823

**Published:** 2013-05-12

**Authors:** Mariarita Dessì, Annalisa Noce, Pierfrancesco Bertucci, Simone Manca di Villahermosa, Rossella Zenobi, Veronica Castagnola, Eliana Addessi, Nicola Di Daniele

**Affiliations:** ^1^Department of Laboratory Medicine, “Tor Vergata” University Hospital, Viale Oxford 81, 00133 Rome, Italy; ^2^Nephrology and Hypertension Unit, Department of System Medicine, “Tor Vergata” University Hospital, Viale Oxford 81, 00133 Rome, Italy

## Abstract

Phospholipids play an essential role in cell membrane structure and function. The length and number of double bonds of fatty acids in membrane phospholipids are main determinants of fluidity, transport systems, activity of membrane-bound enzymes, and susceptibility to lipid peroxidation. The fatty acid profile of serum lipids, especially the phospholipids, reflects the fatty acid composition of cell membranes. Moreover, long-chain n-3 polyunsatured fatty acids decrease very-low-density lipoprotein assembly and secretion reducing triacylglycerol production. N-6 and n-3 polyunsatured fatty acids are the precursors of signalling molecules, termed “eicosanoids,” which play an important role in the regulation of inflammation. Eicosanoids derived from n-6 polyunsatured fatty acids have proinflammatory actions, while eicosanoids derived from n-3 polyunsatured fatty acids have anti-inflammatory ones. Previous studies showed that inflammation contributes to both the onset and progression of atherosclerosis: actually, atherosclerosis is predominantly a chronic low-grade inflammatory disease of the vessel wall. Several studies suggested the relationship between long-chain n-3 polyunsaturated fatty acids and inflammation, showing that fatty acids may decrease endothelial activation and affect eicosanoid metabolism.

## 1. Introduction

Cardiovascular disease is the leading cause of mortality in many economically developed nations accounting for about 30% of all deaths [1] and its incidence is still increasing. Ongoing research aims to investigate and prevent the early development of cardiovascular risk factors such as atherosclerosis, hypertension, dyslipidemia, chronic inflammation, and insulin resistance. 

The beneficial effects of n-3 polyunsaturated fatty acids (n-3 PUFAs) were proved in several observational and experimental studies. The lipid lowering action of n3-PUFAs was detected at the beginning, so these nutrients were used for the treatment of dyslipidemic disorders. Their anti-inflammatory, antithrombotic, antiatherosclerotic, and antiarrhythmogenic effects were observed later. Low-grade chronic inflammation is now recognized as a prominent process in the development of atherosclerosis and coronary heart disease. The induction of inflammation may well provide a link between hyperlipidemia and atherogenesis [2, 3]. 

Atherosclerosis is now considered a “systemic disease” featured by low-grade arterial inflammatory lesions that can develop through the disease progression [4]. 

**Figure 1:**
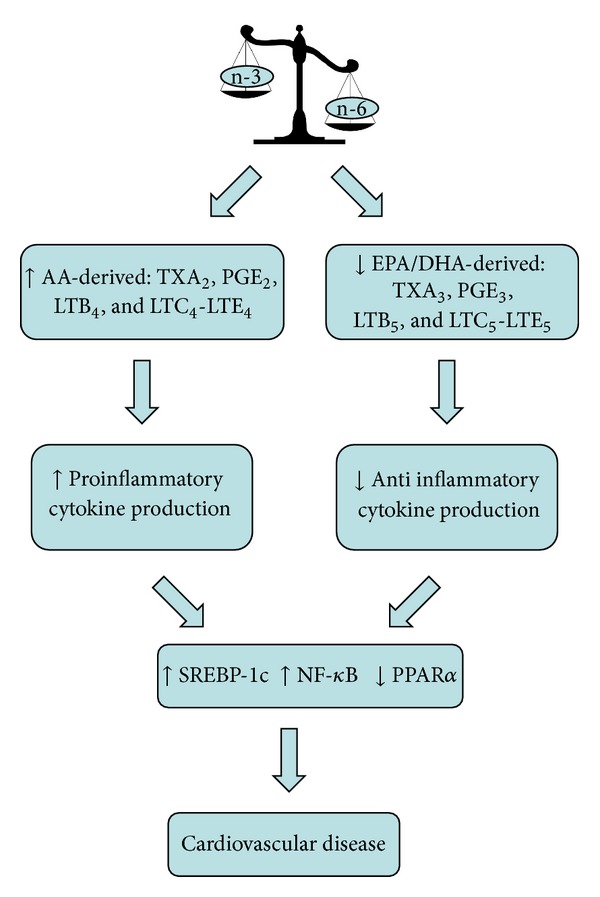
Effects of unbalanced n-6:n-3 dietary fatty acid intake on inflammatory state. EPA: eicosapentaenoic acid; DHA: docasahexaenoic acid; AA: arachidonic acid; PPAR-*α*: peroxisome proliferation-activated receptors-*α*; TX A_2_: thromboxane A_2_; TX  A_3_: thromboxane A_3_; NF-*κ*B: nuclear factor kappa light-chain enhancer of activated B cells; PGE_2_: prostaglandin E_2_; PGE_3_: prostaglandin E_3_; LTB: leukotriene; SREBP-1c: sterol regulatory binding protein 1c.

In physiological conditions, endothelial cells synthesize and release adequate amounts of nitric oxide (NO) and prostaglandins (such as PGE_2_ and PGE_3_) and maintain a downstream balance between pro- and anti-inflammatory molecules. However, in the presence of atherosclerosis this balance disrupts leading towards an increase production of proinflammatory cytokines as interleukins 1, 2, and 6 (IL-1, 2, and 6) and tumor necrosis factor *α* (TNF-*α*), with further progression of the disease [5]. These pro-inflammatory cytokines can induce oxidative stress by enhancing the production of reactive oxygen species (ROS) by monocytes, macrophages, and leukocytes. PUFAs and their eicosanoid derivatives may play a significant role modulating the inflammatory response (Figure 1) [6].

## 2. Metabolism of PUFAs

Unsaturated fatty acids are referred to as PUFAs when two or more double bounds are present. There are two PUFAs families, omega-3 (n-3) and omega-6 (n-6) fatty acids. They differ in location of the last double bond relative to the terminal methyl end of the molecule. The human body can produce almost all fatty acids, except linoleic acid (LA, C18:2 n-6, precursor to the n-6 series of fatty acids) and *α*-linoleic acid (ALA, C18:3n-3, precursor to the n-3 series of fatty acids). These two PUFAs are named “essential fatty acids” because the body cannot synthesize them [7].

Endogenous conversion (elongation and desaturation) of the initial C18 PUFA precursors results in the synthesis of longer-chain counterparts such as eicosapentaenoic acid (EPA) and docosahexaenoic acid (DHA) in the n-3 family and dihomo-*γ*-linoleic acid (DGLA) and arachidonic acid (AA) in the n-6 family [1].

In humans the biochemical pathways converting ALA to EPA and EPA to DHA are limited: 0.2–8% of ALA is converted to EPA (generally more in women) and 0–4% of ALA to DHA [8–12]. Results from pilot studies suggest that ALA conversion is also limited to function as a surrogate for fish consumption [13]. Thus, tissue and circulating EPA and DHA levels are primarily related to their dietary intake. Fish is the major food source of long-chain n-3 PUFAs, including EPA, DHA, and docosapentaenoic acid (DPA), while ALA is a plant n-3 fatty acid mainly found in seeds, nuts, and their oils. Thus, plant sources of n-3 fatty acids cannot currently be considered as a replacement for seafood-derived n-3 PUFAs [14]. This suggests that n-3 fatty acids derived from different sources might have their own specific effects on cardiovascular risk markers. Linoleic acid is thought to decrease the conversion of ALA into EPA and DHA by competing for the n-6-desaturase enzyme [15]. Previous studies showed that genetic variations in this enzyme may be related to cardiovascular disease [16]. Furthermore, because the two fatty acid pathways are mutually exclusive (i.e., n-3 fatty acid cannot become n-6 fatty acid and vice versa), a balanced intake of ALA and LA as precursors or of their longer-chain products EPA, DHA, and AA is required [1]. 

## 3. PUFAs and Cardiovascular Disease

There is a vast amount of epidemiologic evidence of a cardioprotective effect of fish oil-derived EPA and DHA and it was confirmed in randomized controlled trials [17–20]. Several intervention studies, such as the “Gruppo Italiano per lo Studio della Sopravvivenza nell'Infarto miocardico” (GISSI)-Prevenzione trial and the Cardiovascular Health Study, have shown that an increased intake of eicosapentaenoic acid (EPA, C20:5n-3) and docosahexaenoic acid (DHA, C22:6n-3) lowers the risk of coronary heart disease (CHD) [21, 22]. The first GISSI-Prevenzione trial, a randomized open label study in 11324 Italian patients with recent myocardial infarction, demonstrated that patients had a 15% lower combined risk of mortality, nonfatal myocardial infarction, and stroke upon supplementation for 3.5 years with 850 mg*·*day-1 of LC n-3 PUFA. The relative risk of cardiovascular mortality was also decreased by 30% and that of sudden death by 45%. The GISSI-Prevenzione trial demonstrated that a significant protective effect could be obtained with doses much lower than those previously considered necessary for significant beneficial effects [21]. 

The second GISSI-HF study, a randomized double-blind placebo-controlled trial in 6975 Italian patients with chronic heart failure, revealed a moderate decrease in both all-cause mortality admissions to hospital for cardiovascular disease upon supplementation for an average of 3.9 years with 1 g of LC n-3 PUFA daily. Again, the beneficial effects were seen in a population already treated with recommended therapies [23].

The “Japan Eicosapentaenoic acid (EPA) Lipid Intervention Study,” (JELIS) trial, was performed on 18645 Japanese men and women with hypercholesterolaemia treated with statins. Supplementation with 1.8 g EPA daily decreased major coronary events by 19% over 4.6 years. Non-fatal coronary events, rather than CHD death, were decreased [24]. This may be evident in Japanese population where a consistent reduction of CHD is observed probably because of a high background seafood intake [25, 26]. In summary, a modest intake of LC n-3 PUFA significantly decreases the risk of fatal CHD. However, higher doses and longer duration of intervention, as reported for the JELIS study, have the potential to protect from non-fatal CHD events [26]. Presently, there is evidence to suggest that the two LC n3-PUFAs—EPA (eicosapentaenoic acid, 20:5N3) and DHA (docosahexaenoic acid, 22:6N3)—exert pleiotropism (akin to those effects which have been reported for certain cardiovascular drugs) by modulating a range of diverse target mechanisms involved in cardiovascular disease development. [27–30]. Since the appearance of purified forms of DHA in the market in the 1990s, researchers have started to investigate the differential effects of EPA and DHA on cardiovascular health. However, the number of human studies is still limited in this field and the independent effects of EPA and DHA on various cardiovascular outcomes are yet to be firmly established [31]. 

Furthermore, the incorporation of EPA and DHA into the cell membrane influences its organisation, fluidity, and permeability, as well as the activity of transmembrane proteins, including receptors, enzymes, and ion channels. Both EPA and DHA modulate K, Na, and Ca channel activities in myocardial cells, regulating myocyte electrical excitability and contractility [32–34]. These effects are concentration dependent and appear to be mediated by the action of EPA and DHA on membrane fluidity [34], although other mechanisms, such as a direct binding of n-3 LCP to the channel, may be involved [35]. Furthermore, animal studies support the increasing evidence that DHA rather than EPA is preferentially incorporated into the myocardial cell membrane [36]. Collectively, these findings help to explain the antiarrhythmic and heart rate (HR)-lowering effects observed by DHA but not by EPA in humans [37]. In addition, incorporation of DHA into the membrane of cardiomyocytes influences the beta adrenergic system to a greater extent than EPA [38], that is, potentially another important mechanism involved in the hypotensive and anti-arrhythmic action of DHA. Furthermore, DHA incorporation into the membrane of endothelial cells stimulates ATP release from the endothelium, increasing vasodilation by stimulating nitric oxide (NO) release [39]. The induction of NO release, together with the decrease in noradrenaline levels, may also account for the BP-lowering effect of DHA [39]. A recent meta-analysis including thirty randomized controlled trials showed that prolonged fish oil intake may reduce HR, especially in populations with a high baseline HR [40]. In dyslipidemic males and postmenopausal women, this decrease appears to be mediated by DHA rather than EPA [37, 41, 42]. In contrast, no significant effect of either EPA or DHA on HR was observed in healthy males for similar dosage and treatment duration [43]. HR variability (HRV) is a strong predictor of CVD, including sudden cardiac death, arrhythmic CHD, and atrial fibrillation. Fish oils have shown anti-arrhythmic properties in animal studies [36], and several clinical and epidemiological studies have reported an association between HRV and n-3 LCP blood cell levels and/or fish oil intake [44–46].

The relationship between ALA and cardiovascular disease is less clear. Short-term trials (6–12 week) in healthy individuals showed no or low effect of a daily intake of 1.2–3.6 g of ALA on blood lipids, LDL-oxidation, lipoprotein (a), and apolipoproteins A-I and B [8, 47]. Long-term treatment with high-dose ALA (40 g/daily) showed a beneficial effect by reduction of body weight and blood LDL/total cholesterol ratio [47, 48]. Previous evidence favored recommendation for modest dietary consumption of ALA (2-3 g daily) in the primary and secondary prevention of CHD [19]. The recently published case-control study by Campos et al. [49] shows a strong inverse associations between ALA status-intake and nonfatal MI. Recent data support the assumption that ALA may protect against atherosclerosis [50, 51]. However, data from two recent epidemiologic studies suggest that high tissue ALA is related to an increased rather than decreased risk of fatal cardiovascular events [52] and sudden death [53]. Also, n-3 PUFAs may interfere with CVD with other physiological effects: the reduction of triglyceride (TG) synthesis is well recognized [54]. Moreover, the reduction of hepatic VLDL synthesis may contribute to this protective effect reducing the availability of fatty acids for triglyceride synthesis due to the decreased “*de novo*” lipogenesis (DNL), increasing the beta-oxidation of fatty acids, reducing the delivery of nonesterified fatty acids to the liver, reducing the activity of TG synthesis by hepatocytes, and finally increasing the hepatic synthesis of phospholipids [54–59]. 

In both experimental models and human studies, reduction of DNL appears to be prominent [54–60]. A different effect was reported in some studies between EPA and DHA: both EPA and DHA produced a significant reduction of TGs and LDL-cholesterol, raising effects of fish oil [61, 62]. EPA showed antiplatelet and anti-inflammatory properties [63, 64].

Recently, Buckley and coworkers [65] showed that a four-week supplementation with DHA significantly reduced TG levels in normolipidemic human subjects by 22%, while EPA decreased TG levels by 15% without reaching statistical significance. In another four-week interventional study, both EPA and DHA reduced postprandial TG without affecting fasting TG levels in healthy human subjects [66]. However, EPA and DHA seemed to reduce triglyceridaemia to the same extent when given for a long-enough period [60, 61, 67–72]. 

Studies in humans show that an average intake of n-3 PUFAs 3-4 g/daily decreases serum triacylglycerol concentration by 25–30% in a dose-dependent manner. The same intake does not affect total cholesterol but increases LDL cholesterol by 5–10% and HDL cholesterol by 1–3% [73]. 

The increase in LDL cholesterol is mainly due to a rise of the larger and the potentially less atherogenic LDL particles [74].

Hypertension (AH) and dyslipidemia (DLP) are the most important and frequent risk factors for CVD in the general population, with a prevalence of 50–80% according to different authors [75–79]. The prevalence of DLP is 40% among untreated hypertensive patients. On the other hand almost half of the patients with increased total cholesterol (TC) have systo-diastolic hypertension [80–83].

Modifications of erythrocyte-membrane fatty acids (FA) composition are an early indicator of the development of AH and lipid disorders [79]. Thus, modifications of erythrocyte-membrane FA are fairly subtle indicators of lipid metabolism pathology which manifest themselves much earlier than changes in plasma lipoproteins. Modifications of fatty acids composition of the cell membrane lipid matrix play an important role in the AH pathogenesis. Changes of the fatty acids (FA) composition with decrease of essential PUFAs may result in increase of membrane microviscosity, activation of pro-inflammatory eicosanoids synthesis and increased sensitivity of the smooth muscle cells in artery walls to the influence of vasoconstrictors [84]. That is the reason of the great interest in the study of the spectrum of erythrocyte lipids in hypertensive dyslipidemic patients. 

Deficiency of n-3 PUFA is a distinctive feature of the modification of erythrocyte-membrane FA in hypertensive dyslipidemic patients. In particular Novgorodetseva and coworkers observed a significant reduction of 22:5n-3 and 22:6n-3 [79]. Deficiency of endogenous n-3 PUFAs may lead to changes in physicochemical properties of cell membranes, activation of the synthesis of proinflammatory and vasoconstrictive eicosanoids, and finally induction of a systemic inflammatory syndrome [84]. All these conditions may favor the induction and progression of atherosclerosis [85]. Thus, structural and functional changes in erythrocyte cell membranes develop in hypertension as a part of the common systemic disorder of lipid metabolism. The FA disorganization of the cell membrane may cause the development of hyperlipidemia and the progression of hypertensive disease [74]. Thus, an important role of FA in the pathogenesis of cardiovascular disease seems clear. 

A large part of the cardioprotective actions of LC n-3-PUFAs is likely to be mediated by the vascular endothelium [28, 86–88]. This thin monolayer of cells plays a central role in cardiovascular homeostasis and function via the production of a range of potent autocrine and paracrine biochemical mediators that control the tone of vascular smooth muscle cells [31, 89, 90]. Vascular endothelium is also the site for the inception, progression, and clinical manifestations of atherosclerosis [91–93].

**Figure 2:**
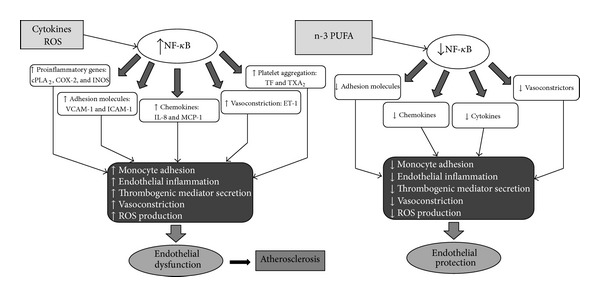
Relationship between inflammation, atherosclerosis, and n-3-PUFA. NF-*κ*B: nuclear factor kappa light-chain enhancer of activated B cells; ROS: reactive oxygen species; cPLA2: cytosolic phospholipase A2; COX-2: cyclooxygenase-2; i-NOS: inducible nitric oxide synthase; VCAM-1: vascular cell adhesion protein 1; ICAM-1: intercellular adhesion molecule-1; IL-8: interlukin-8; MCP-1: monocyte chemoattractant protein-1; TF: tissue factor; ET-1: endothelin-1; TX A_2_: thromboxane A_2_.

**Figure 3:**
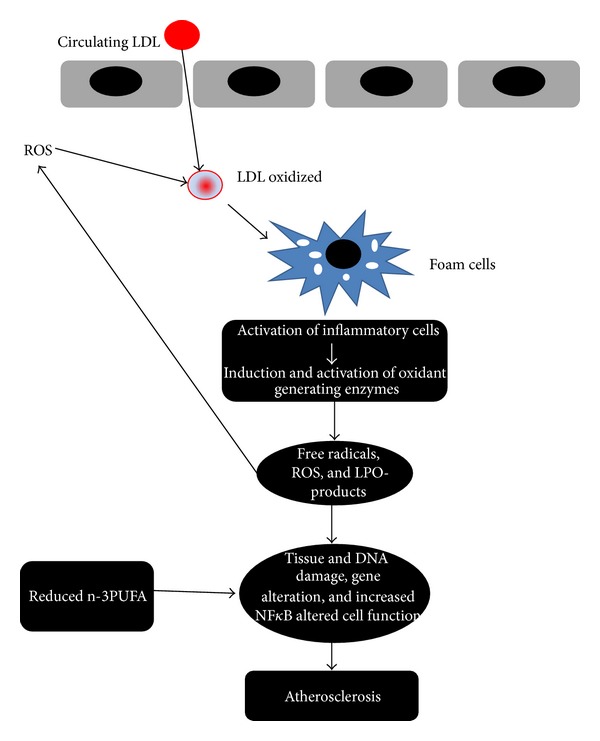
Factors and cells involved in the development of atherosclerosis. ROS: reactive oxygen species; LPO-products: lipo-peroxidation products; NF-*κ*B: nuclear factor kappa light-chain enhancer of activated B cells.

Multifactorial endothelial dysfunction induced by DLP, toxins, cigarette-smoking, and so on may be the early event in the development of atherosclerosis. The endothelium becomes “proadhesive” and induces an increased adhesion of circulating monocytes that subsequently infiltrate the arterial intima. At this level endothelial cells or macrophages release reactive oxygen species that oxidize circulating LDL into oxidized-LDL and create the “lipid streak” [94]. In the evolution from the lipid streak to the atherosclerotic plaque, numerous cytokines that cause infiltration of smooth muscle layer by leukocytes and fibroblasts and promote platelet adhesion are involved in turn [2] (Figures 2 and 3).

During the migration over the vessel wall, monocytes are exposed to various proinflammatory stimuli that contribute to their differentiation into macrophages. After the absorption of lipoproteins, these macrophages become “foam cells.” Other macrophages induce a local inflammatory compartment within the vassel wall, that is, continuously powered by cytokines, activated T-helper cells and scavanger receptor activators. The inhibition of this self-maintaining process may reduce atherogenesis as demonstrated in mutant mice deficient for macrophage colony stimulating factor (M-CSF) or macrophage migration inhibitory factor (MIF) [95, 96]. In more advanced lesions, cell debris, apoptotic cells, and free cholesterol particles accumulate in the lipid-rich necrotic core of the plaque, are sometimes separated from the blood only by a thin fibrous cap. The number of inflammatory cells within the lesion is strongly associated with the risk of rupture of the so-called “vulnerable plaque.” At last, the process may propagate intraluminal thrombosis, thereby distal ischemia of heart or brain tissue [97, 98].

Through their positive antithrombotic, lipid-lowering, proendothelial functional activities, n-3 PUFAs play an important role on the mechanisms of atherosclerosis. In fact supplementation of N-3 PUFAs improves flow-mediated arterial dilatation by an increased endothelial synthesis of nitric oxide [86, 99–111]. Several, although not all, clinical trials have also found that consumption of n-3 PUFAs lowers circulating markers of endothelial dysfunction, such as E-selectin, vascular cell adhesion molecule-1, and intracellular adhesion molecule-1 [74, 112, 113]. Thus, normalization of endothelial function could partly mediate the protective effects of n-3 PUFA against CVD. A recent randomized clinical trial established the role of PUFAs on plaque stability [114]. Patients with carotid artery atherosclerosis were randomized to take placebo, fish oil (n-3 PUFAs), or seed oil (n-6 PUFAs). Subjects treated with n-3 PUFAs showed higher EPA and DHA concentration, reduction of monocytes and macrophages, and a thicker fibrous cap compared to controls and group treated with n-6 PUFAs, all changes that can enhance the stability of atherosclerotic plaques. By contrast, increased assumption of n-6 PUFAs did not affect carotid plaque fatty acid composition or stability. Plaques stability may explain the reductions in nonfatal and fatal cardiovascular events, associated with increased n-3 PUFAs intake [114]. The triglyceride-lowering, antiatherogenic, antithrombotic, and anti-inflammatory effects of n-3 PUFAs were clearly demonstrated in human studies. Conversely, their anti-arrhytmogenic effect was showed only in vitro and in animal experiments, as confirmation in humans has been limited by the absence of reliable physiological measures or biomarkers to quantify antiarrhythmic potential [115]. The main effects exerted by PUFAs in cardiovascular diseases are summarized in Table 1.

## 4. PUFAs and Inflammation

Inflammation is a physiological response to tissue trauma or infection, but leukocytes, which are the effector cells of the inflammatory process, have powerful capabilities of tissue remodelling. Thus, the passage of leukocytes from the bloodstream into inflamed tissue is tightly regulated to ensure their precise localization. Recruitment of circulating neutrophils into the tissue stroma occurs during the early phases of inflammation. In this process, peptide agonists of the chemokine family are assumed to provide a chemotactic stimulus capable of supporting the migration of neutrophils across vascular endothelial cells, through the vessel wall and out into the tissue stroma. Although an initial chemokine stimulus is essential for the recruitment of flowing neutrophils by endothelial cells, stimulated by the inflammatory cytokine (TNF-*α*), transit across the endothelial monolayer is regulated by additional stimuli [116].

A new step in the neutrophil recruitment process that relies upon a lipid-mediated signal to regulate the migration of neutrophils across endothelial cells was recently described. This signal is supplied by the metabolism of arachidonic acid into the eicosanoid prostaglandin-D2 (PGD_2_) by cyclooxygenase (COX) enzymes. This step in the neutrophil recruitment process was revealed when the EPA was used as an alternative substrate for COX enzymes, leading to the generation of prostaglandin-D_3_ (PGD_3_) [117–120]. This alternative eicosanoid inhibited the migration of neutrophils across endothelial cells competing for the PGD_2_ receptor [121, 122]. PGD_2_ signalling is subordinate to the chemokine-mediated activation of neutrophils, but without the sequential delivery of this signal, neutrophils fail to penetrate the endothelial cell monolayer. The ability of dietary EPA to inhibit this process reveals an unsuspected level of regulation in the migration of inflammatory leukocytes. This may contribute to clarify the interactions between PUFAs and the inflammatory system and direct research on novel therapeutic agents that target the inflammatory system with greater affinity and/or specificity than dietary supplements of n-3-PUFAs [116].

Acute inflammation and its healing are essential processes for tissue protection. The inflammation healing mechanisms are of main interest, and in recent years, new endogenous anti-inflammatory and proresolving lipid mediators generated from PUFAs (lipoxins, resolvins, protectin, and maresin) were uncovered [123]. Lipid mediator metabolomics of self-resolving inflammatory exudates recently highlighted a new family of potent anti-inflammatory and proresolving mediators. Serhan and coworkers identified families of novel bioactive mediators derived from arachidonic acid (AA-derived lipoxins), eicosapentaenoic acid (EPA-derived E-series resolvins), and docosahexaenoic acid (DHA-derived D-series resolvins, protectin, and maresin) [124–135]. These lipid mediators promote resolution through enhanced clearance of apoptotic PMNs, chemokines, cytokines, and microbial products by macrophages [136, 137].

N-3 PUFAs may act through several mechanisms, for instance, by preventing conversion of the n-6 PUFA arachidonic acid to pro-inflammatory eicosanoids and by their conversion to potent anti-inflammatory mediators such as resolvins [124–126, 138].

A possible biological mechanism underlying the beneficial effects of LC n-3 PUFAs on inflammation and endothelial function is that they may compete with n-6 fatty acids for prostaglandin and leukotriene synthesis at the cyclooxygenase and lipoxygenase levels. LC n-3 PUFAs from fish or fish oil modulate prostaglandin metabolism by increasing the active vasodilator and inhibitor of platelet aggregation prostaglandin E3, the weak platelet aggregator and vasoconstrictor thromboxane A3, and the weak inducer of inflammation leukotriene B5. Otherwise, the production of thromboxane A2, a potent platelet aggregator and vasoconstrictor, and leukotriene B4, an inducer of inflammation and a powerful inducer of leukocyte chemotaxis and adherence is reduced [139]. Another suggested mechanism is that LC n-3 PUFAs may bind reactive oxygen species because of their multiple double bonds and lead to a decreased production of hydrogen peroxide. Hydrogen peroxide is a critical activator of the nuclear factor-*κ*B system of transcription factors that controls the coordinated expression of adhesion molecules and of leukocyte-specific chemoattractants upon cytokine stimulation [140]. He and coworkers [141] found independent inverse associations of LC n-3 PUFAs and nonfried fish with IL-6, CRP and MMP3. The relevance of these biomarkers of inflammatory and endothelial activation in the atherogenic process is well recognized. Previous investigations suggest that both CRP and IL-6, two systemic inflammatory markers, are independent predictors of CVD and may play an important role in atherogenesis [142]. In addition, MMP3 is suggested as an independent prognostic factor in stable coronary artery disease [143]. Several lines of evidence support the main role of MMPs in plaque stability [144].

Soluble intercellular adhesion molecule-1 (sICAM-1) is thought to be a key factor in the adherence of monocytes to the endothelium and subsequent transmigration into the intima (Figure 2). The role of sICAM-1 in the pathogenesis of inflammation and atherosclerosis has been confirmed in experimental models [145]. Previous investigations also indicate that sICAM-1 is an independent predictor of CVD apart of other traditional risk factors [146]. He et al. [141] observed that fried fish but not non-fried fish consumption was significantly inversely related to sICAM-1. This finding is not expected since the frying process may reduce the content of LC n-3 PUFAs and produce trans-fatty acids. One possible explanation is that fried fish consumption may be a marker of relatively unhealthy lifestyle; those who had high fried fish consumption were more likely to suffer from dyslipidemia and were likely to be under treatment with medications (e.g., statins), which may lower sICAM-1 [147]. However, the inverse association between fried fish intake and sICAM-1 level remained significant when patients taking cholesterol-lowering medications were excluded. Anyway, further investigation is warranted [148]. 

LC n-3 PUFAs are believed to affect inflammatory processes mainly through two pathways: endothelial activation and changes in eicosanoid production, or a combination of the two [28]. Two hypotheses may define these molecular mechanisms. The first, predicated on the observation that n-3 PUFAs regulate the transcription of endothelial cell inflammatory genes by downregulating the activity of the nuclear factor-kB, predicts changes in the levels of adhesion receptor and chemokine expression after supplementation with EPA [149–151]. The second hypothesis proposes that upon endothelial cell activation, EPA may compete with the n-6-PUFA arachidonic acid (AA; 20:4n-6) for cyclooxygenase enzymes (COX1 and COX2) after both fatty acids are released from membrane phospholipids by endogenous phospholipases [152]. The main effects exerted by PUFAs in inflammatory processes are summarized in Table 1.

## 5. Conclusions

A large amount of investigations suggest, the cardioprotective effects of LC n-3 PUFAs EPA and DHA intake in human subjects, because of their lipid lowering, hypotensive, anti-arrhythmic, and anti-thrombotic properties. Moreover, studies performed in the last twenty years showed heterogeneous effects of different n-3 PUFAs on various cardiovascular outcomes, which may be of paramount relevance in primary and secondary prevention of cardiovascular disease.

Recent in vitro investigations as well as clinical studies also demonstrated that LC-n3-PUFAs significantly interact with inflammation-related mechanisms, such as endothelial activation, modification of eicosanoid metabolism, and resolution of the inflammatory process. 

Low-grade chronic inflammation is present in several diseases and is characterized by abnormal circulating levels of pro- and anti-inflammatory cytokines. N-3 PUFAs may modulate inflammation, that is, suggested by the reduction of plasma inflammatory cytokines (TNF-*α*, Il-6) and inflammatory markers as high sensitive C reactive protein, observed after the intake of EPA and DHA. Low-grade chronic inflammation plays a key role in the induction and progression of atherosclerosis and consequently of cardiovascular disease. Taking into consideration the pleiotropic nature of their actions, we suggest that dietary intake of LC n-3 PUFAs may lead to improvements in cardio-metabolic health parameters of their antioxidant, anti-inflammatory, and antiarrhytmic actions.

## Figures and Tables

**Table 1:** PUFAs and cardiovascular disease.

PUFA effects	
(1) Anti-inflammatory	(a) ↓NF-*κ*B activation
	(b) EPA and DHA compete with AA for COX & 5-lipo-oxygenase enzymatic sites
	⇓
	Reduce the production of IL-1, IL-6, and TNF-*α*
	(c) ↑Anti inflammatory eicosanoids

(2) Cardiac energetic	(a) ↑ATP generation
	(b) ↓O_2_ consumption
	(c) ↓Sarcoplasmic reticulum calcium concentration
	⇓
	Maintain normal mitochondrial function

(3) Antiarrhythmic	(a) ↑Ca^2+^/Mg^2+^ ATPase activity
	(b) Inhibit fast voltage-dependent Na^+^ channels (*I*_Na_)
	(c) Inhibit L-type Ca^2+^ channels (*I*_Ca,L_)
	⇓
	Membrane stabilization
	(d) Reduced automaticity
	(e) Increased relative refractory period

(4) Hemodynamics	(a) Improved endothelium-independent and dependent vasodilatation
	(b) ↓ET-1
	(c) ↑NO
	⇓
	Improved endothelium dysfunction

(5) Ventricular remodeling and fibrosis	(a) ↑PPAR*γ* → ↑adiponectin
	⇓
	Attenuates ventricular remodeling

(6) Vascular	(a) ↓Platelet aggregation via ↓TXA_2_
	(b) ↓VCAM-1, ELAM-1, ICAM-1
	(c) ↓monocyte endothelial adherence via ↓PAF

EPA: eicosapentaenoic acid; DHA: docosahexaenoic acid; AA: arachidonic acid; ET-1: endothelin-1; NO: nitric oxide; PPAR-*γ*: peroxisome proliferation-activated receptors-*γ*; TX A_2_: thromboxane A_2_; VCAM-1: vascular cell adhesion protein 1; ELAM-1: endothelial leukocyte adhesion molecule-1; ICAM-1: intercellular adhesion molecule-1; PAF: platelet activating factor; COX: cyclooxygenase.

## Abbreviations

n-3 PUFAs: n-3 polyunsaturated fatty acids
NO: Nitric oxide
PGE_2_; PGE_3_: Prostaglandins
IL-1: Interleukins 1
IL-2: Interleukins 2
IL-6: Interleukins 6
TNF-*α*: Tumor necrosis factor *α*
ROS: Reactive oxygen species
LA: Linoleic acid
ALA: *α*-Linoleic acid
EPA: Eicosapentaenoic acid
DHA: Docosahexaenoic acid
DGLA: Dihomo-*γ*-linoleic acid
AA: Arachidonic acid
DPA: Docosapentaenoic acid
CHD: Coronary Heart Disease
HRV: Heart Rate Variability
Tgs: Triglyceride
AH: Arterial Hypertension
DLP: Dyslipidemia,
TC: Total cholesterol
FA: Fatty acids
M-CSF: Macrophage colony stimulating factor
MIF: Migration Inhibiting Factor
PGD_2_: Prostaglandin D2
COX1-COX2: Cycloxygenase 1-2
TXA: Thromboxane A
sICAM-1: Soluble intercellular adhesion molecule-1.
